# Biomimetic learning of hand gestures in a humanoid robot

**DOI:** 10.3389/fnhum.2024.1391531

**Published:** 2024-07-19

**Authors:** Parthan Olikkal, Dingyi Pei, Bharat Kashyap Karri, Ashwin Satyanarayana, Nayan M. Kakoty, Ramana Vinjamuri

**Affiliations:** ^1^Department of Computer Science and Electrical Engineering, Sensorimotor Control Lab, University of Maryland, Baltimore, MD, United States; ^2^Department of Computer Systems Technology, City Tech at City University of New York, New York, NY, United States; ^3^Department of Electronics and Communication Engineering, Tezpur University, Assam, India

**Keywords:** MediaPipe, hand kinematics, kinematic synergies, biomimetic robots, human robot interaction, bioinspired robots, sign language recognition, hand gestures

## Abstract

Hand gestures are a natural and intuitive form of communication, and integrating this communication method into robotic systems presents significant potential to improve human-robot collaboration. Recent advances in motor neuroscience have focused on replicating human hand movements from synergies also known as movement primitives. Synergies, fundamental building blocks of movement, serve as a potential strategy adapted by the central nervous system to generate and control movements. Identifying how synergies contribute to movement can help in dexterous control of robotics, exoskeletons, prosthetics and extend its applications to rehabilitation. In this paper, 33 static hand gestures were recorded through a single RGB camera and identified in real-time through the MediaPipe framework as participants made various postures with their dominant hand. Assuming an open palm as initial posture, uniform joint angular velocities were obtained from all these gestures. By applying a dimensionality reduction method, kinematic synergies were obtained from these joint angular velocities. Kinematic synergies that explain 98% of variance of movements were utilized to reconstruct new hand gestures using convex optimization. Reconstructed hand gestures and selected kinematic synergies were translated onto a humanoid robot, Mitra, in real-time, as the participants demonstrated various hand gestures. The results showed that by using only few kinematic synergies it is possible to generate various hand gestures, with 95.7% accuracy. Furthermore, utilizing low-dimensional synergies in control of high dimensional end effectors holds promise to enable near-natural human-robot collaboration.

## 1 Introduction

The marvel of evolution is evident in the versatility of human hands. Years of bipedal life and opposable thumb have promoted the extensive usage of hands for grasping, reaching and dexterous manipulation. These appendages can delicately cradle a butterfly, skillfully handle the brush for creating a masterpiece and firmly grip a hammer. Within the human hand lies a complex arrangement of joints, tendons, muscles, all connected meticulously by the nerves. The coordination of these elements allows for dexterity and precision enabling us to express a wide spectrum of gestures and manipulate objects with complex surface. A simple kinematic model of the human hand has more than 20 degrees of freedom (DoF) making it an extremely difficult problem to be replicated in robots. Despite its simplicity, the study of human hand movements has been a significant area of research for more than three decades and both researchers and roboticist have been actively trying to address the challenge of replicating the prowess of human hand dexterity in a robot.

With the advancement of technology, traditional devices for interaction with computers are replaced with more natural communication approaches such as oral communication and body language. Among these two methods, the functional means of natural communication is body language interaction, with hands being the most effective non-verbal means of communication. During interpersonal communication, the impact of our messages is often enhanced by following hand gestures. For instance, certain gesture requires the synchronous movement of all four fingers and thumb whereas others require individual finger movements. Consequently, the range of hand gestures for communication extends from simple to complex hand movements. By integrating hand gestures as an interactive tool and the ability to classify them into meaningful symbols or values, there is a potential to develop more intuitive human-robot interaction (HRI) and human-computer interaction (HCI) interfaces that can potentially assist individuals with motor impairments. Hand gesture-based interaction systems have thus become a magnetic area of research since its introduction in 1970s. A diverse array of human computer interactive systems have been developed using hand gesture control such as sign language recognition (Rastgoo et al., 2020), improving motor skills (Cai et al., 2018) and user guide interactive applications (Indriani Harris and Agoes, 2021).

The human hand, with its 27 bones, 29 muscles and over 20 DoFs, is a marvel of engineering. Because of this intricate anatomy, there is a huge possibility to execute one movement such as picking up a bottle of water, through various coordinated combinations of muscles and joints. Moreover, there are multiple ways to accomplish the same movement, underscoring the remarkable flexibility and adaptability of the hand’s complex structure. But how does the human brain navigate through the vast possibilities of movement to control the human hand? Modularity hypothesis introduced by Bernstein (1967) was able to address most of the challenges of the large DoFs and thereby the large number of redundant choices for performing a simple task. The neuroscientific reasoning for this strategy is the finding that, despite the complexity of the human hand, fewer variables can adequately account for most of the variation in patterns of human hand configurations and movements. For instance, consider the act of picking up a bottle of water. While there are countless combinations of muscle contractions and joint rotations that could accomplish this task, the central nervous system (CNS) does not default to a brute force strategy. Instead, it chooses an approach that activates specific groups of muscles and joints in a coordinated pattern. Bernstein in his modularity hypothesis called these variables as synergies. Synergies, thus, act as building blocks, simplifying the control of the vast DoFs of the human hand. Understanding these synergies can provide insights to decoding brain-hand communication, understanding motor disorders, and potentially incorporating them into robotic control algorithms. Inspired from modularity hypothesis, several researchers have investigated synergies obtained from different parts of the body such as kinematic synergies (Grinyagin et al., 2005; Freitas et al., 2006), muscle synergies (Weiss and Flanders, 2004; Muceli et al., 2010; Santello et al., 2013; Tagliabue et al., 2015), force synergies (Santello and Soechting, 2000), and dynamical synergies (Pei et al., 2022). Kinematic synergies obtained from finger joint kinematics and muscle synergies extracted from muscle movements have gained much popularity among the others. Here, in this study, we would be focusing on kinematic synergies obtained from joint kinematics while executing hand movements.

How are synergies established in humans, and can we apply these synergy learning techniques to robot to mimic human learning? To explore such questions, in this study, we employed American Sign Language (ASL) as a common ground to bridge the gap between human and robot learning. ASL’s complex hand movements require motor planning and synergy formation. This approach allows us to investigate the neural mechanisms during the formation of new hand gestures. In the process of learning a new motor activity, there are two distinct stages – (a) the identification and reinforcement of motor synergies necessary for performing the new task, and (b) weakening of synergies, as explained in Latash (2010). By utilizing ASL as a test bed, attempts to execute complex hand gestures can potentially reveal identifiable motor synergies. Our goal is to replicate this motor learning in a robot, providing insights into the neural mechanisms of motor planning and execution along with their limitations. Building on the evidence from previous studies (Vinjamuri et al., 2010; Patel et al., 2018; Pei et al., 2019; Olikkal et al., 2022a), which leverage electroencephalography (EEG), electromyography (EMG), and hand joint kinematics to identify neural mechanisms of motor planning and motor execution, we aim to understand and replicate these synergies in a robotic system. Burns et al. (2017) contributed to this understanding by developing a soft hand exoskeleton – a form of human-machine teaming – embedding motor synergies for assisting or rehabilitating individuals with hand disabilities.

**Figure 1:**
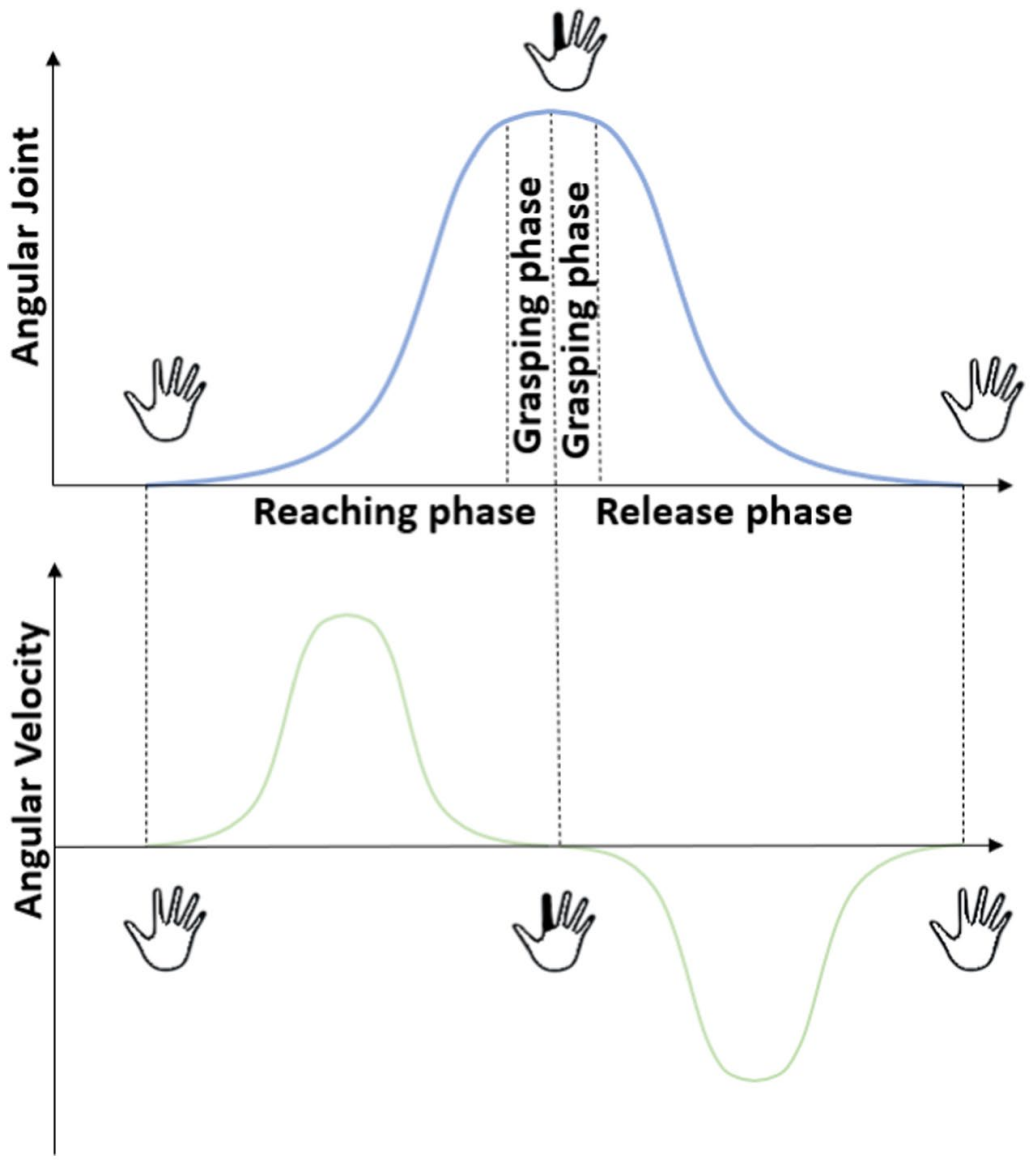
Movement decomposition phases of reaching, grasping and release is illustrated. The angular joint profile and angular velocity profiles of one joint at different phases can be observed here.

Research study by Jarque-Bou et al. (2019b) adopted a phase-based approach, decomposing the movement profile into distinct stages: reaching, grasping/manipulation and release (Figure 1) in an attempt to understand how the CNS controls and coordinates the hand when aiming to execute a targeted movement. The joint angular velocity profiles of these three phases collected from joint recording devices such as CyberGlove are well represented and investigated in Vinjamuri et al. (2010), Jarque-Bou et al. (2019a,b), and Olikkal et al. (2022a,b). These studies reinforce that when a subject attempts to reach a target from initial reference position, a progressive increase in angular velocity profile can be observed in the reaching phase. This reflects the rapid movement of the hand towards the target. During the grasping phase the velocity transitions to a steady state highlighting the smooth and controlled adjustment of finger position to securely grasp the object. Finally, the release phase exhibits a gradual decline in velocity, reaching back to the initial reference state as elaborated in Jarque-Bou et al. (2019b). Upon close observation, as indicated in Jarque-Bou et al. (2019b), joint angular velocity profile was collected from sensors that corresponds to metacarpophalangeal, interphalangeal and proximal interphalangeal joints of each finger from the CyberGlove.

Focusing on a single joint, it becomes evident that the angular velocity profile for any movement can be effectively split similarly into a start, target and return phases where the target phase represents the flexion or extension of the joint to achieve a desired state along its DoF and the start and return phases represents the initial state of that joint (shown in Figure 1). During the execution of hand gestures, the joint angular velocity profiles for these joints consistently exhibits a Gaussian curve as the target state is reached. Therefore, we draw inspiration from the above studies that incorporate this observation of a Gaussian curve made by each joint during any sort of hand movements.

Breakthrough in technology have encouraged the development of numerous robotic devices aiming to mimic human arm and finger movements by observing the kinematic patterns. These coordinated kinematic patterns are usually extracted and embedded into these devices to aid in performing activities of daily living. Several promising rehabilitative exoskeletons using kinematic synergies are detailed in Jarrassé et al. (2014). However, there have been a limited exploration to understand the efficiency of kinematic synergies in humanoids. Hauser et al., in their study (Hauser et al., 2011) was able to use few non-linear kinematic synergies from lower body to transform the balance control challenge into a linear problem for a humanoid robot during slow movements. Alexandrov et al. (2017) validates the human inspired kinematic synergy as a potential candidate for balance control among the group of control concepts. To the best of our knowledge, apart from our previous study (Olikkal et al., 2023), there has been a notable absence of exploration into humanoid robots performing upper limb movements using kinematic synergies. Unlike the humanoid, Pepper (Pandey and Gelin, 2018), through this study we attempt to provide an analysis of using biologically inspired human kinematic synergies on a humanoid robot for dexterity.

The field of hand gesture recognition has undergone significant development. Hand gesture recognition based on the extracted feature and different recognition approaches are described in Indriani Harris and Agoes (2021). Traditional motion capturing sensors and devices are now replaced with more intuitive frameworks that simplify gesture recognition applications. An example of this shift is seen in Google’s open-source framework, MediaPipe, which offers multiple machine-learning solutions, replacing conventional methods. Among the several solutions provided by MediaPipe for vision tasks such as object detection, face detection and gesture recognition, we opted for hand landmark detector in this paper. MediaPipe hand landmark detector enables to identify the hand landmarks in an image. This model thus allows one to apply graphic effects over the hand image and localize key hand regions. Employing such a framework for gesture recognition not only helps identify hand landmarks in challenging environments and backgrounds, but also enables adequate focus and attention in deriving joint movement kinematics and postures.

The following is a synopsis of this paper’s key contributions.

1. Compared to the research study that uses RGB and depth camera for capturing hand grasps (Ficuciello et al., 2013; Devineau et al., 2018), our research advances the existing pipeline (Olikkal et al., 2023) by applying the proposed framework to specifically recognize 33 hand gestures in American Sign Language. Not only does this study increases the complexity of our preliminary hand gesture database but also highlights the practical applicability and robustness of the framework in real-world scenarios.
2. Utilizing a limited number of synergies, our pipeline successfully reconstructs all 33 hand gestures in the dataset. This work extends our preliminary results (Olikkal et al., 2023), by exploring the role of kinematic synergies in reconstructing hand gestures on a humanoid robot on a broader hand gesture database, thus demonstrating the versatility and efficiency of our approach.
3. Furthermore, we propose an online model that demonstrates real-time translation of identified hand gestures to a humanoid robot, enabled by kinematic synergies. This contribution serves as a foundational step towards achieving seamless interaction between humans and robots through gesture-based communication, highlighting the potential for more intuitive and natural HRI.

## 2 Methods and analysis

### 2.1 MediaPipe Framework

**Figure 2:**
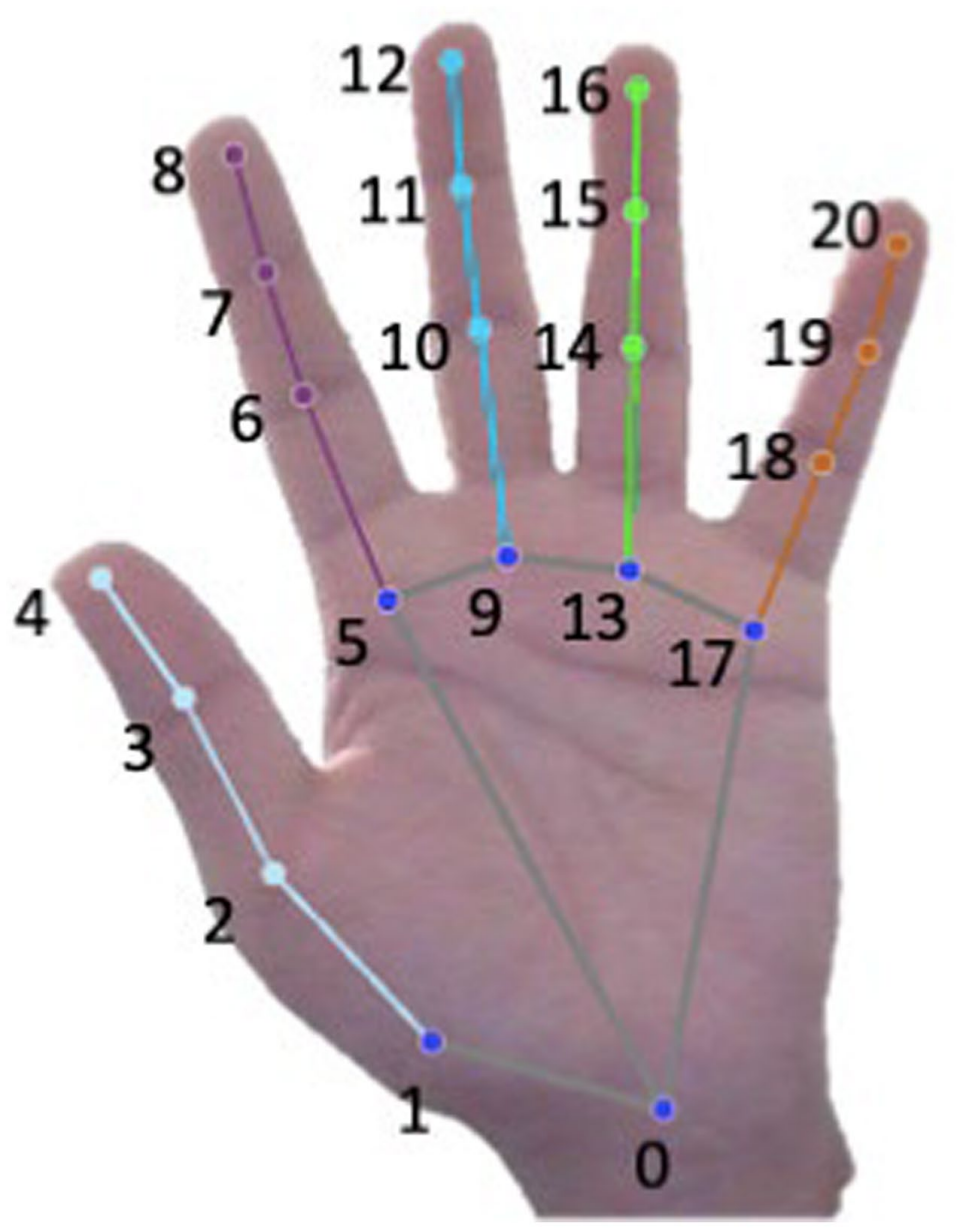
Twenty-one hand-knuckle landmarks obtained from MediaPipe, and their corresponding anatomical areas are illustrated here. Each dot represents the joints and line represents the Euclidean distance from each joint. Corresponding labels in the illustration are 0. Wrist, 1. CMC-Thumb, 2. MCP-Thumb, 3. IP-Thumb, 4. TIP-Thumb, 5. MCP-Index Finger, 6. PIP-Index Finger, 7. DIP-Index Finger, 8. TIP-Index Finger, 9. MCP-Middle Finger, 10. PIP-Middle Finger, 11. DIP-Middle Finger, 12. TIP-Middle Finger, 13. MCP-Ring Finger, 14. PIP-Ring Finger, 15. DIP-Ring Finger, 16. TIP-Ring Finger, 17. MCP-Pinky, 18. PIP-Pinky, 19. DIP-Pinky, 20. TIP-Pinky.

As outlined by Zhang et al. (2020), a real-time hand gesture recognition system has been developed using a single RGB camera which can predict the skeleton of a human hand. MediaPipe hand landmark detector leverages two modes – a palm detector model and a hand landmark model. The palm detector model focuses on identifying the palm by analyzing the entire image and produces an image with an oriented binding frame of the hand. The hand landmark model takes in the cropped binding box image as input and through regression returns 3D hand key points on the image. The model outputs 21 key points on the 3D hand-knuckle skeletal image on the hand. Each of the identified landmark is composed of distinct relative x, y, and z coordinates where x and y are normalized by image width and height whereas z represents the depth of the landmark. In Figure 2, these points are illustrated with dots representing joint and lines indicating Euclidean distances between them. The Euclidean distance measure is calculated between each landmark, serving as a condition for identifying various hand gestures based on the arrangement of these points.

### 2.2 The humanoid robot – Mitra

This study incorporated a humanoid robot, Mitra (Invento Research Inc., Plano TX). Mitra is a custom-built robot equipped with 21 DoFs, distributed among its various components. The configuration includes 5 DoFs for each finger, 1 DoF for the wrist, 1 DoF for the elbow, 2 DoFs for each shoulder, along with 1 DoF for the head and 2 DoFs for the base. Mounted on the top of the head is an RGB camera with a resolution of 1,280 × 720 pixels, enabling real-time image and video capture. To facilitate the grasping of heavy objects, additional support is provided for the digits on the right hand. Mitra is also equipped with a LiDAR system for mapping its surroundings. The robot offers multiple connectivity options, including voice commands, web interfaces, touch screen, joysticks, and scripts. In this study, a scripting method was adopted for hand gesture modeling and communication with Mitra.

#### 2.2.1 Hardware architecture of Mitra hands

Mitra employs three distinct types of motors, each providing varying degrees of torque, strategically placed at different joints to facilitate movement. The primary motor, which generates the maximum torque, is located at the shoulder joint, responsible for shoulder flexion and extension. A second motor with moderate torque is positioned at the elbow joint, facilitating elbow flexion and extension. The third type comprises servo motors, which are dedicated to controlling finger movements.

For precise control of the hand digits, each hand is equipped with five servo motors, corresponding to the five digits of each hand. Each servo motor operates through three lines: a ground, power, and control. These motors are governed by hierarchical network of microcontrollers. The control line, influenced by the voltage supplied from the parent microcontroller, dictates the degree of rotation of the servo motor. This rotation mechanism retracts a cable that loops through digit of Mitra, thereby controlling the flexion and extension. A higher voltage from the microcontroller results in a greater rotation of the servo motor, causing significant retraction of the cable and consequently, more pronounced digit flexion. Conversely, a lower voltage results in lesser rotation and causes minimal digit movement, either in terms of flexion or extension from a flexed position.

#### 2.2.2 Software architecture of Mitra hands

The software component of Mitra’s control system is based on an asynchronous socket communication between Mitra and a commanding system. This setup allows the commanding system to act as a controller transmitter and Mitra as a receiver. Commands specifying the required joint movements are transmitted from the transmitter system to Mitra.

An internal processor within Mitra is responsible for interpreting these commands. This internal system parses the incoming messages and dispatches the appropriate instructions to various microcontrollers that control the different motors in Mitra. Each microcontroller, upon receiving its command, adjusts the voltage supplied to its associated motor to achieve the desired degree of flexion or extension.

### 2.3 Experiment

For this study, two models were developed – an offline model and online model. The offline model involves the creation of a database containing ASL hand gestures using MediaPipe. The online model involves subjects posing ASL hand gestures to Mitra and Mitra mimicking the same gestures in real-time using kinematic synergies. The development of the offline model utilized MATLAB and Python, while the online model was exclusively created using Python.

#### 2.3.1 Offline model

**Figure 3:**
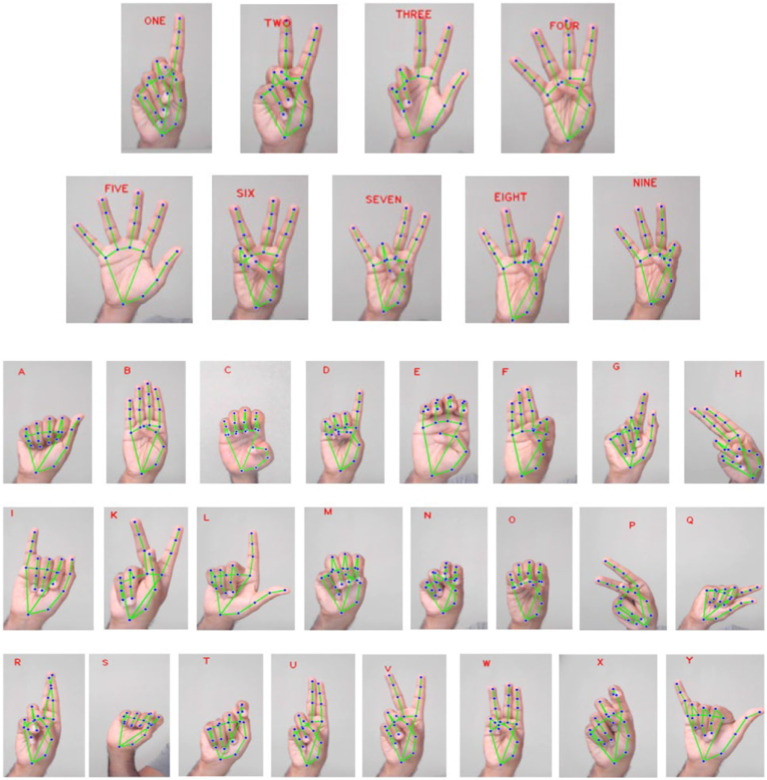
The dataset consists of 9 static ASL number gestures and 24 static ASL alphabet gesture recognized through MediaPipe is shown here.

A database of ASL hand gestures was created which included 24 static alphabets and 9 static numbers as shown in Figure 3. The hand gestures were detected using the MediaPipe hand landmark detection model from an RGB camera mounted on Mitra when gestures were presented. After identifying the landmarks, based on the Euclidean distance of the x and y coordinates from the wrist, the open and closed state of the thumb and the open, half-open, and closed state of the index, middle, ring and pinky fingers were determined. Based on the open, half-open and closed state of the digits, different hand gestures were identified. Alphabet “J” and “Z” were not included in this study because of their dynamic nature. All 33 static ASL hand gestures were shown to Mitra from an initial reference posture of a relaxed idle open palm hand posture.

#### 2.3.2 Online model

In this model, five subjects (4 male and 1 female) with mean age of 27 ± 4.4 years and no prior upper limb movement disorders, were recruited to perform hand gestures. The experiment, along with the data collection, conformed to an approved protocol by the Institutional Review Board (IRB) at the University of Maryland Baltimore County. Written informed consent to participate in this study was provided by the subjects following university guidelines.

The experimental setup involved subjects sitting comfortably in front of a screen displaying various ASL hand gestures. Each subject was instructed to choose any ten hand gestures from the screen and show the gestures using their dominant hand in front of the RGB camera mounted on Mitra. During the experiment, the MediaPipe hand landmark model was employed to detect and annotate the hand gestures posed by the subjects, with the results of the recognized gesture displayed on a separate screen. Once all ten selected hand gestures were demonstrated, Mitra autonomously selected the appropriate hand gesture from a pool of kinematic synergies developed in the offline model and mimicked the hand gestures posed by the subjects.

## 3 Derivation of synergies in the offline model

### 3.1 Synthetic joint angular velocities

From the hand gestures presented to Mitra, the end postures of each gesture were transformed into joint angular velocities using a Gaussian function, as expressed in the Eq. (1) with respect to the initial reference posture:


(1) $$\varphi =\frac{1}{\sqrt{2\pi \sigma^{2}}}e^{-\frac{1}{2\sigma^{2}}{\left(x-\mu \right)}^{2}}$$

**Figure 4:**
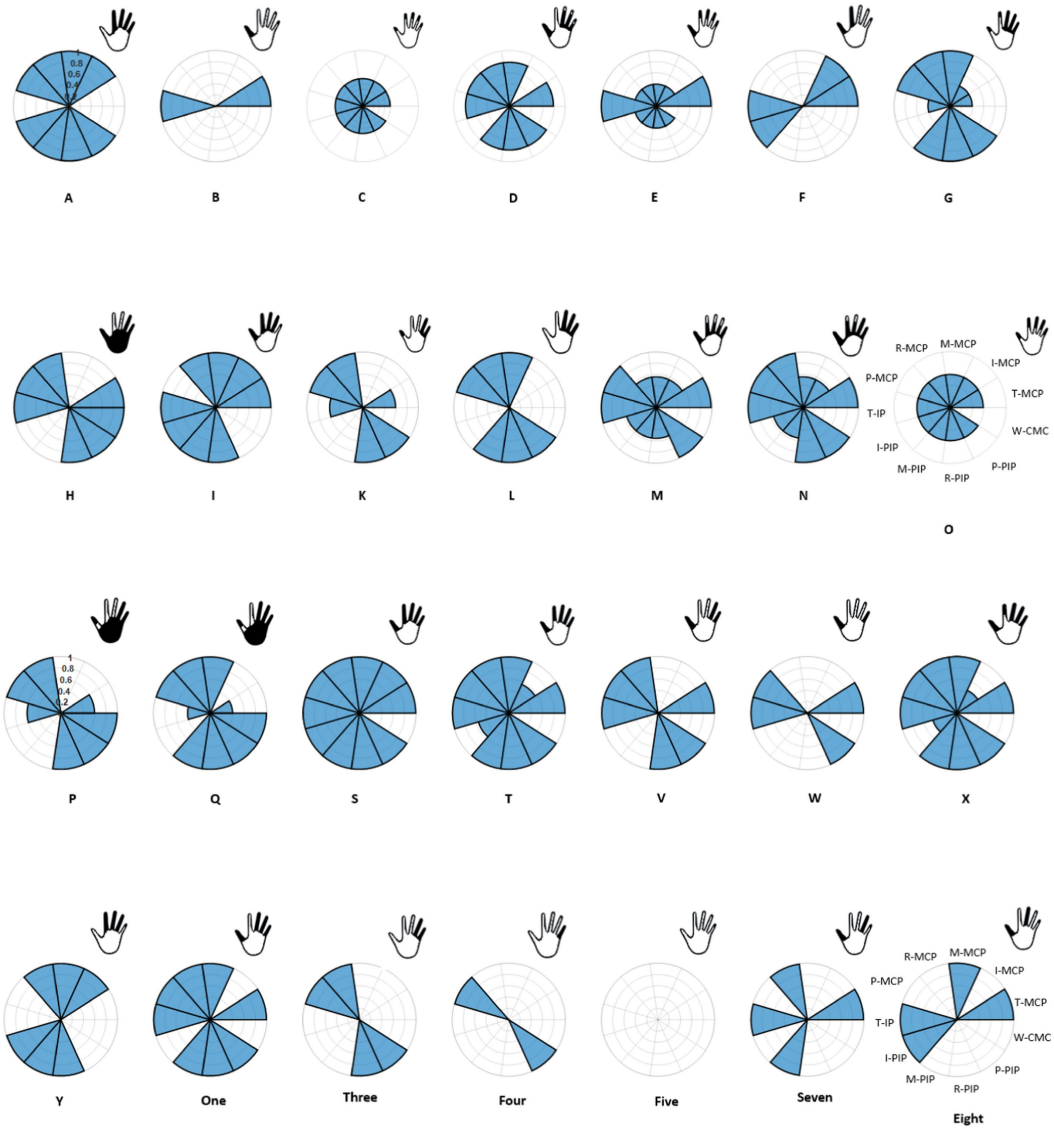
Conversion of 28 hand gestures to joint velocities are illustrated here. Each pie represents one hand gesture with 11 joints and each sector represent one sensor. Those joints that are activated are shaded in blue and their corresponding finger representation is shown in the top right posture.

Here, φ is the generated Gaussian curve, σ is the standard deviation and μ is the mean of x formed from a sample of 100,000 randomly generated data points. A total of 11 such velocity profiles were created corresponding to ten joints of the hand and one joint of the wrist – metacarpophalangeal (MCP) and interphalangeal (IP) joints of the thumb and MCP and proximal interphalangeal (PIP) joints of the other four digits. An additional carpometacarpal (CMC) joint of the wrist was included to indicate those movements which included wrist. Thus, for each ASL static hand gestures considered, the corresponding joint angular velocities for the 11 joints were computed. This process involved transforming the observed hand gestures into sets of angular velocities of the specified joints. For each joint that was involved in forming the end posture of the hand gesture, its corresponding joint was represented with the Gaussian function expressed in Eq. (1). Joints that made partial contributions towards the end postures were represented with lower amplitude of the Gaussian function (e.g., hand gesture X in Figures 3, 4).

In the context of ASL alphabet and numbers, gestures with similar representation were excluded from the ASL number gestures. Specifically, the removal included gestures for two, four, six, and nine, as their corresponding representation in ASL alphabet gestures are V, B, W and F, respectively. Given that only the x and y coordinates of the hand landmark were considered in this study, gestures with similar representations but differing in z-axis orientation were adjusted by modifying the velocity profile of CMC. This adjustment pertained to gestures such as U and H, G and Q, K and P. Two exceptions were made – for gesture 4, instead of thumb flexion, pinkie was flexed, and H gesture was represented as V with CMC profile. Analysis of Figure 4 reveals that certain representation of the hand vector appear similar, specifically in the cases of C and O, as well as D and One gestures. However, a closer examination of the joint angular velocity profiles indicate variations in amplitude, distinguishing these gestures from each other. Despite the visual similarities in the hand vector representations, the unique patterns in the joint angular velocities provide a more detailed and distinctive characterization of each gesture.

### 3.2 Synthetic kinematic synergies

Joint angular velocities corresponding to the end postures of 10 hand gestures were synthetically generated using the Gaussian function. For gestures involving specific joint flexion, the relevant joint out of 11 were represented with Gaussian function. Once the joint angular velocities were generated for the selected 28 hand gestures, the dataset was split into training set consisting of 20 gesture tasks and testing set with 8 gesture tasks. Following the methodology in Vinjamuri et al. (2010), an angular velocity matrix was constructed using the gestures in the training set such that each of the 11 joints were cascaded one after the other. Thus, each row of the angular velocity matrix represents one gesture.

Leveraging the insights from our previous works (Olikkal et al., 2022a,b) it was observed that time-variant synergy models yielded best results, hence for this study, we opted for the time-variant synergy model. In case of time-variant models, a time-varying pattern is produced by combining synergies with time-varying scaling coefficients. A time-varying synergy signifies the synchronized activation of a group of joints at a particular time for each joint. Diverse patterns can be generated by adjusting the coefficients and temporally shifting different synergies. Mathematically, this can be expressed as shown in Eq. (2)


(2) $$M\left(t\right)=\overset{N}{\underset{i=1}{\sum }}A_{i}.S_{i}\left(t-t_{i}\right)$$

Here, $M\left(t\right)$ represents the generated time-varying pattern, N is the total number of synergies, $A_{i}$ is the scaling coefficient for the i th synergy, $S_{i}\left(t-t_{i}\right)$ denotes the *i*th synergy shifted in time by $t_{i}$ .

Principal component analysis (PCA) was applied on the cascaded velocity matrix to derive PCs that capture the maximum variance. Following our prior works (Olikkal et al., 2022a,b), to identify the optimal number of PCs, we selected those PCs the accounted for 0.98 (98%) of total variance using the Eq. (3) expressed as


(3) $$\frac{\lambda_{1}+\lambda_{2}+\ldots +\lambda_{m}}{\lambda_{1}+\lambda_{2}+\ldots +\lambda_{n}}\geq 0.98$$

Where λ represents the magnitude of the corresponding PCs and m represents the optimal number of synergies out of n available synergies. When this fraction reaches to 0.98 the corresponding m identifies as the optimal number to be chosen. These chosen m PCs were termed as synthetic kinematic synergies. It was observed that combining six synthetic kinematic synergies contributed to around 98% of the total variance.

### 3.3 Reconstruction of hand gestures using, *l*_1_-minimization

The joint angular velocities of the 8 gesture tasks grouped under the testing set were reconstructed using the derived synthetic kinematic synergies. These synergies obtained can serve as templates for decomposing hand movements. Investigations from Vinjamuri et al. (2010) implies that the CNS strategically utilizes a small number of synergies to generate movement. Following the methodology in Vinjamuri et al. (2010), a matrix was formed, termed as bank, which contains the row vectors of the synthetic kinematic synergies and their five possible shifts. Consequently, for any given hand gesture and an existing bank of template synergies, multiple coefficients can be found to represent the gesture. Given that the CNS utilizes only a limited set of kinematic synergies and a small number of coefficients for executing hand gestures, this current problem of identifying limited coefficients can be conceptualized as an *l*_1_-minimization problem as described in Vinjamuri et al. (2010). It can be formulated as an optimization problem aimed at identifying the sparsest coefficients for hand movement generation expressed in Eq. (4) as


(4) $$\mathrm{Minimize}∥c{∥}_{1}+\frac{1}{l}∥cB-\mathrm{gesture}{∥}_{2}^{2}$$

**Figure 5:**
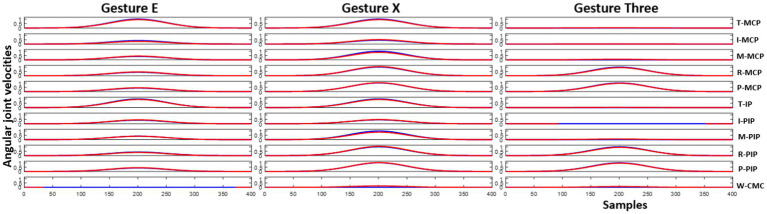
Joint angular velocities of the hand gesture E, X and three are illustrated here. Blue line represents the joint angular velocities of the test hand gesture generated through the Gaussian function and red line represents the reconstructed joint angular velocities using the kinematic synergies and coefficients.

Here, $∥c{∥}_{1}$ represents the $l_{1}$ norm, B is the synthetic kinematic synergy bank and l is the regularization parameter. Solving this optimization problem results in a set of coefficients that efficiently reconstruct the hand gesture using the provided template kinematic synergies. Hand gestures grouped under the testing data can thus be reconstructed by combining the coefficients found through Eq. (4) with the kinematic synergies from the bank. Figure 5 illustrates three different randomly selected hand gestures – E, X, and three.

Upon identification of the coefficients, gestures grouped under the testing sets were reconstructed. Reconstruction error between the synthetic angular velocities ( $M_{i}$ ) and the reconstructed patterns ( X ) using time-variant synergies were determined as followed in Eq. (5).


(5) $$\mathrm{err}=\frac{\sum_{i}{\left(M_{i}-X\right)}^{2}}{\sum_{i}M_{i}^{2}}$$

### 3.4 Translating hand gestures to Mitra

The reconstructed patterns of gestures, along with the six synthetic kinematic synergies and the test hand gestures were further translated to Mitra. To facilitate this translation, a moving average function and a scaling coefficient were applied to map these patterns to the joints of Mitra. Continuous input from the reconstructed patterns and test data was provided to Mitra during the gesture execution. This process ensured that Mitra mimicked and executed the hand movements based on the reconstructed patterns obtained from synthetic kinematic synergies.

## 4 Real-time hand gesture using online model

In the offline model, synthetic kinematic synergies were extracted from a pool of 28 ASL gestures. When a subject pose any of the selected hand gestures from the available ASL hand gestures, the MediaPipe hand landmark detection model is employed to recognize the hand gestures using Euclidean distance measures. Based on the hand gesture identified, an joint angular velocity profile is generated. Six synthetic kinematic synergies are selected from the offline model such that the training set excludes the current hand gesture. Time-shifted versions of these six synthetic kinematic synergies were obtained from the offline model. Using convex optimization, limited coefficients are determined to accurately reconstruct the hand gesture with the chosen six synthetic kinematic synergies. The reconstructed gesture is then mapped to Mitra’s hands through a mapping function effectively mimicking the hand gesture posed by the subject. The entire process occurs in real-time, facilitated by the integration of MATLAB Engine and Python to convert the offline model to an online framework.

## 5 Results

### 5.1 Offline model

From the 11 synthetic joint angular velocities generated through the Gaussian function, the end postures of the 28 hand gestures were derived. Six synthetic kinematic synergies were then extracted from these hand gestures, which were grouped under the training set using PCA. A 28-fold cross-validation was performed to reduce the variance in the performance of time-variant synergy model. On average, across all the 28-fold cross-validation trials in the training set, the first synergy accounted for approximately 65.6% of the total variance, the second synergy contributed to 80% of the variance and the first six synergies together captured about 98.5% of the variance on an average. This aligns with observations in Patel et al. (2015), where the first synergy accounted for 50% of the variance and incorporating additional synergies increased the variance. This indicates that from the synthetic joint angular velocities for 28 hand gestures, a relatively small set of synergies could effectively represent the joint movements associated with the hand gestures.

**Figure 6:**
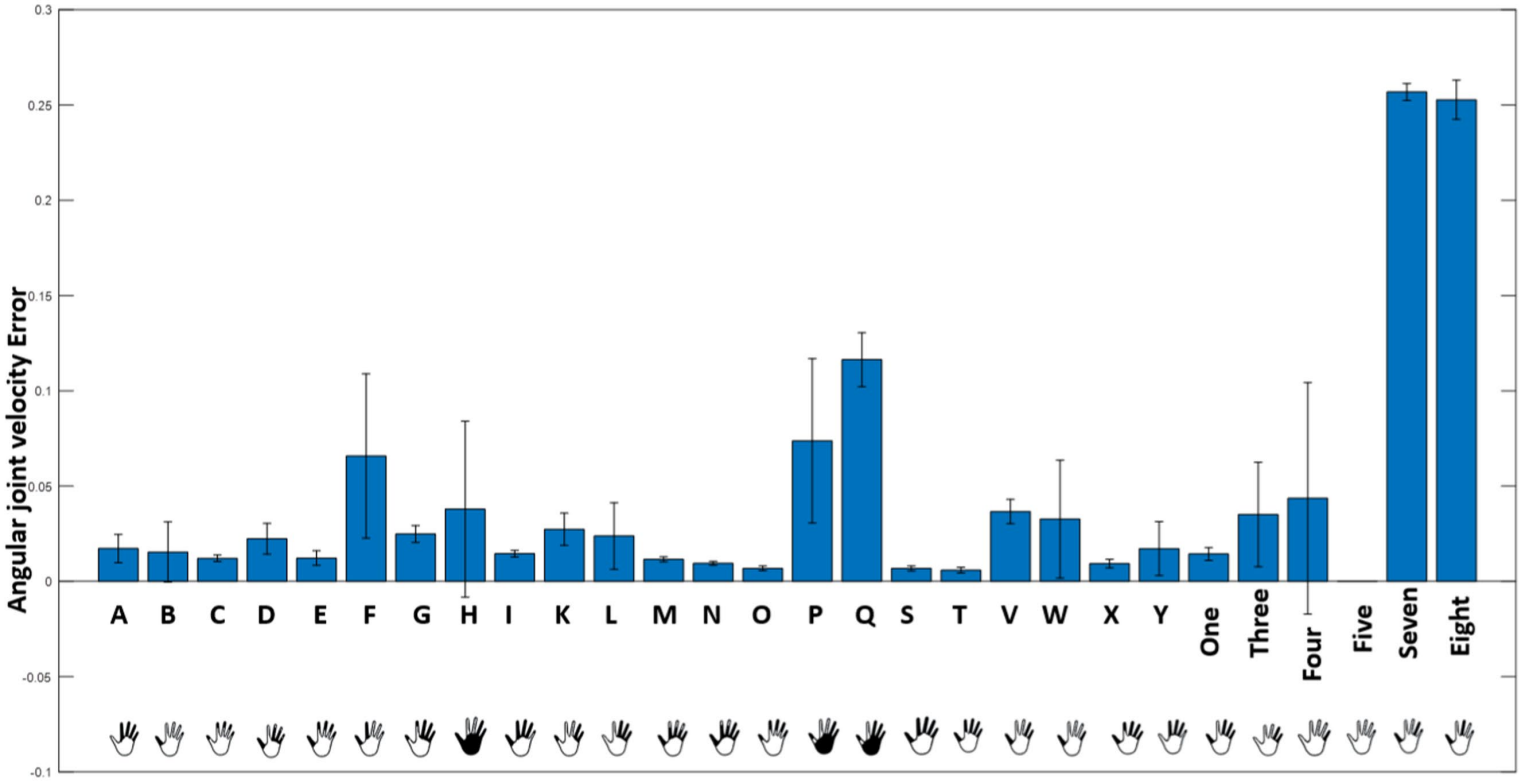
Mean reconstruction error obtained while reconstructing the 28 hand gestures using synthetic kinematic synergies is illustrated here.

**Figure 7:**
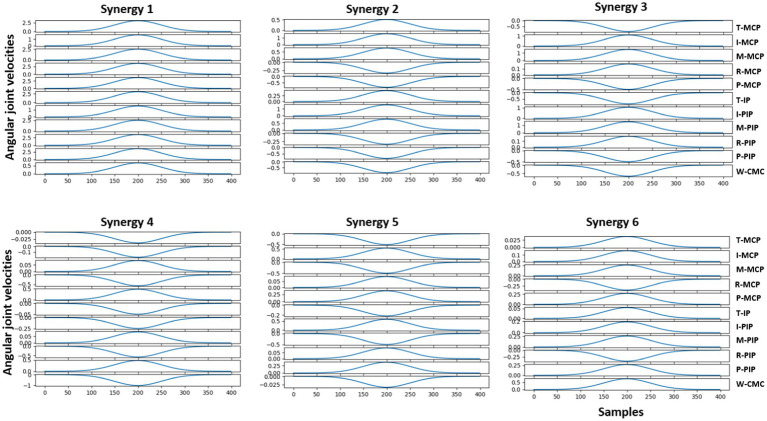
Joint angular velocities of first three synthetic kinematic synergies of the 11 joints extracted from the training data is illustrated here. Here, T-Thumb, I-Index, M-Middle, R-Ring, P-Pinky, MCP-Metacarpophalangeal, PIP- Proximal Interphalangeal, IP-Interphalangeal, CMC-Carpometacarpal joints.

**Figure 8:**
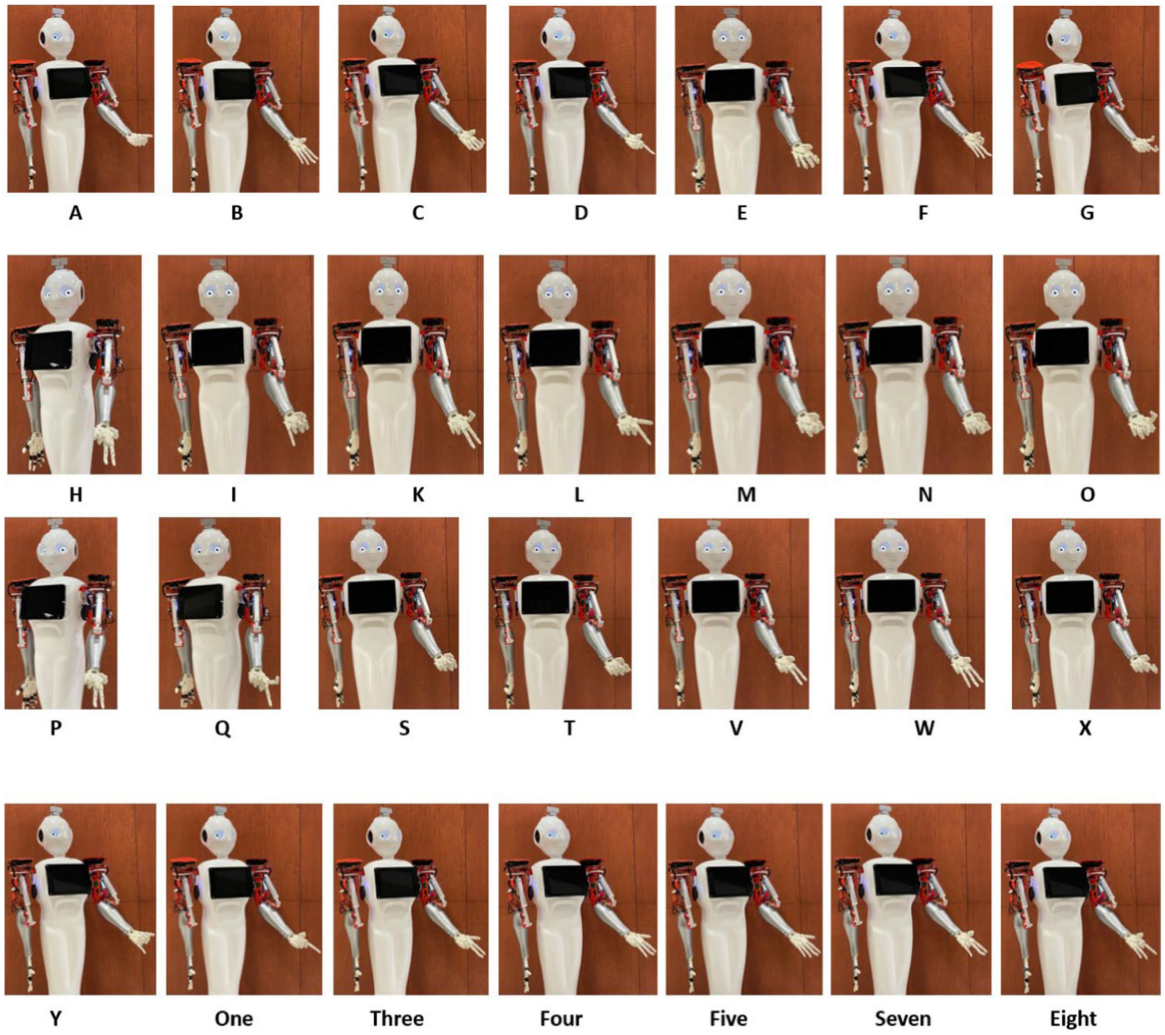
Reconstructed hand gestures represented in Mitra using first six synthetic kinematic synergies for all the ASL hand gestures used in this study.

Reconstruction of the end posture of the test hand gestures were performed using synthetic kinematic synergies. By employing 28-fold cross-validation, each hand gesture appeared in the testing set eight times. As mentioned previously, the reconstruction of the test hand gesture patterns was compared with the synthetically generated joint angular velocities for that hand gesture using the least squared error. Figure 5 represents the trajectories of the joint angular velocities for three distinct hand gestures – E, X, and three. Remarkably, the optimization algorithm successfully identified coefficients that, when combined with kinematic synergies, resulted patterns exhibiting minimal difference across the 11 joint movements for hand gestures under the test data. Figure 6 represents the reconstruction error of 28 hand gestures reconstructed using synthetic kinematic synergies across all 28-fold cross-validation runs. It can be noted that the angular velocity reconstruction error of the gestures Seven is notably higher followed by Eight and Q gestures. In contrast, all the other hand gestures were reconstructed with more than 90% accuracy. Thus, on an average, 28 hand gestures were reconstructed with an accuracy of 95.7%. Synthetic kinematic synergies extracted from the training set as shown in Figure 7 were also mapped to Mitra. In the process of mapping these synergies, Mitra hands were initially flexed to 50%, serving as an initial reference posture. The movements were then mapped such that any value above 50% was interpreted as flexion, while any value below 50% was considered as extension. This mapping strategy ensured that synergies were accurately translated and applied to Mitra’s joints during execution. The reconstructed patterns of the 28 hand gestures were mapped to Mitra as shown in Figure 8.

In Figure 7, it is evident that the joint angular velocity profile of the first synergy primarily involves flexion with varying amplitudes, while the profiles of the other synergies contain both flexion and extensions with smaller amplitudes. This reinforces the results mentioned in Patel et al. (2015) and our previous studies that the synergy with the maximum variance may potentially account for the majority of the movement profile followed by the next synergy with the second maximum variance. By combining these synergies as a weighted linear combination, the end postures of the hand gestures were successfully reconstructed.

### 5.2 Online model

**Figure 9:**
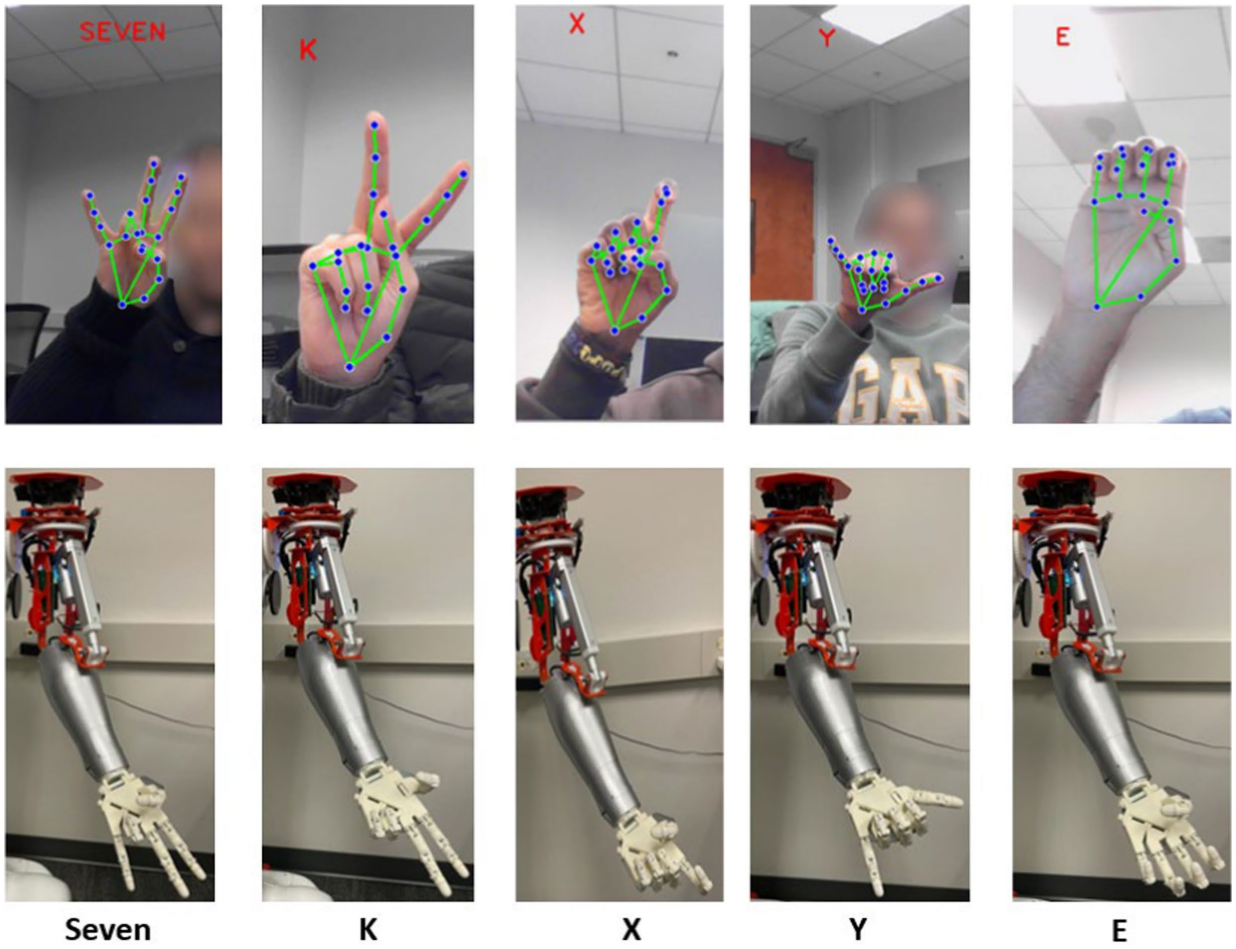
5 subjects performing real-time ASL hand gesture detection using MediaPipe from Mitra’s RGB camera is shown here. These gestures are translated to Mitra instantly. It can be noted that under complex background with various illumination, the model was able to detect the hand gestures.

In this model, five subjects were asked to perform ten different ASL hand gestures from the screen which displays all the static 33 hand gestures. Each hand gesture was posed by the subject using their dominant hand in front of the RGB camera on Mitra. MediaPipe hand landmark model allowed for the accommodation of hand size variability, ensuring that individuals with diverse hand dimensions can effectively interact with Mitra. The developed model was able to detect various hand gestures in complex background with diverse illuminations, objects, and patterns as seen in Figure 9. From the hand gestures captured by the RGB camera, the end posture of each gesture was converted to joint angular velocities utilizing the Gaussian function for each of the 11 joints. Synthetic kinematic synergies extracted from training set, excluding the posed hand gesture, were selected. As mentioned before, a bank of shifted synthetic kinematic synergies was created from the extracted six kinematic synergies. Using the convex optimization, a limited set of coefficients were determined. The shown hand gestures were reconstructed using these coefficients and the bank of kinematic synergies. Subsequently, these reconstructed hand gestures were translated into Mitra’s joints to mimic the posed hand gesture. Figure 9 represents the different hand gesture posed by 5 subjects during the real-time gesture recognition process.

## 6 Discussion

Numerous investigations (Tresch et al., 2006; Steele et al., 2013; Santuz et al., 2017; Taborri et al., 2017) have been done to show that synergies are not merely a mathematical representation but rather an efficient tool for comprehending how the CNS organizes motor control and coordination. As a result of such studies, promising results (Artemiadis and Kyriakopoulos, 2006; Artemiadis et al., 2010; Hocaoglu and Patoglu, 2012; Cunha et al., 2016; Lunardini et al., 2016) have led to the use of synergies in several applications including robotics.

This paper presents the exploration of synthetic kinematic synergies derived from human-inspired joint angular velocities generated through a Gaussian function. This approach involves capturing end postures of 24 static ASL alphabet and 9 static ASL number gestures. Five hand gestures that were similar in the alphabet and number gestures were excluded from ASL number gestures. An exception was made for the number gesture ‘4’. Rather than flexing the thumb, pinkie finger was flexed to indicate the gesture ‘4’. Since this study involved only flexion and extension of the fingers, ‘U’ and ‘R’ were excluded. The reason for this is because of the adduction and abduction of the index and middle finger to represent them which is out of the scope of this study. But to accommodate more information for CMC flexion, we made another exception to represent gesture “H” as gesture “V” with CMC flexion.

Thus, these synthetic joint angular velocities are used to derive synthetic kinematic synergies, which were later used to reconstruct new hand gestures. To the best of our knowledge, this is one of the first attempts to extract synthetic kinematic synergies from Gaussian-function generated joint angular velocities apart from our previous study (Olikkal et al., 2023). The choice of using Gaussian functions to represent finger flexion is motivated by the observation that the joint angular velocity profile of a simple finger flexion tends to exhibit a bell-shaped, Gaussian-like velocity profile, effectively capturing the three phases of movements as indicated in Figure 1. By applying this Gaussian function to express flexion of fingers, end postures of 28 hand gestures were generated. By using only a few synergies, hand gestures grouped under the testing tasks were reconstructed in the offline model. The reconstructed and the recorded patterns of the hand gestures under the test data were then mapped into Mitra.

Of the different mapping approaches such as joint-joint mapping, cartesian space mapping (Gioioso et al., 2013; Rosell and Suárez, 2015), joint-cartesian mapping (Meattini et al., 2020) and object-based mapping (Gioioso et al., 2019) as elaborated in Salvietti (2018), in this study we adopt the joint-joint mapping approach which has shown promising results (Ciocarlie and Allen, 2009; Rosell and Suárez, 2015) for a direct relationship between the corresponding joints of the human hand and Mitra’s hand. Such joint-joint mapping approaches allows for high mapping capabilities for the hand gesture data used for this study.

Although a simple mapping of the 21 landmarks found from MediaPipe Hand landmark detector (Zhang et al., 2020) or learning from human demonstrations such as behavioral cloning (Torabi et al., 2018; De Coninck et al., 2020) to a robot can essentially perform the hand gestures. But in this study, we attempt to show that using only six synergies to control the 21 landmarks, 33 hand gestures can be executed. In approaches that involve learning from human demonstrations, one of the key challenges is to convert the human hand motion into robot hand motion. Assuming a humanoid with 21 joints, the 21 hand landmarks need to be translated to the robot to obtain a single hand gesture. Similarly, through behavioral cloning, motion retargeting of the 21 joints from human demonstration to robot needs to be performed to achieve the same hand gesture. Moreover, multiple demonstrations of the same hand gesture need to be recorded and provided to the robot to learn that gesture. To collect these demonstrations of the same gesture requires long hours of intense human effort from setting the angle of multiple cameras to verifying and eliminating outlier data manually.

Approaches like deep learning and transfer learning have shown remarkable prowess in various applications, including hand gesture recognition and robotic control. For example, Zhan (2019) utilized deep 2D convolutional neural networks to classify 9 different hand gestures in real-time using data augmentation strategies. Oyedotun and Khashman (2017) employed stacked denoising autoencoders and convolutional neural networks to classify and recognize 24 static ASL hand gestures. Wu et al. (2021) collected images of 10 hand gestures using RGB and the 21 joint points of hand using Leap Motion, implementing an effective transfer learning method to classify these gestures. Using MediaPipe hand landmark detection, Peral et al. (2022) was able to develop an efficient and reliable deep learning approach for hand gesture recognition real-time that was translated to a robot. Safavi et al. (2024) provides a comprehensive overview of the various methods in human robot interaction, control, and coordination.

Despite these advancements, our study presents a novel approach using a synergy-based model to control hand gestures with increased efficiency. By utilizing only six synergies to manage the 21 landmarks, our method can execute 33 distinct hand gestures. This approach significantly reduces the complexity and human effort involved in gesture replication. Unlike traditional methods, which requires multiple demonstrations and extensive data collection, our model can achieve accurate gesture execution with only one demonstration. This minimizes the need for extensive human intervention and data collection, thereby increasing overall efficiency. By leveraging a subset of the 21 landmarks, our approach proves a practical and efficient alternative to deep learning and transfer learning methods. Thus, this study extends the application of synergies in hand gesture control, highlighting their potential to simplify and improve robotic hand gesture replication.

In this study, Mitra has only one DoF for each digit, totaling 5 DoF for the 5 digits. The MCPs of these 5 digits can be controlled and based on the flexion, the MCPs move in a gradient fashion accounting for the flexion of PIPs and Distal Interphalangeal joints (DIPs). Each of the 10 hand gestures were demonstrated only once at the RGB camera of Mitra for the offline model and the end postures are generated from the MediaPipe framework. Kinematic synergies extracted from the training data (in Figure 7) of the generated joint angular velocities were then translated to the humanoid using the mapping function. However, since kinematic synergies are bipolar in nature, meaning they have both positive and negative activation potential accounting for flexion and extension, the initial reference state of Mitra hands were adjusted to accommodate for this property. Thus, the MCPs of Mitra were set to 50% flexed as the initial reference state. Upon mapping the selected six synergies to Mitra, joints below this reference were indicated as negative activation potential while those above the reference state were indicated as positive activation potential. These mapped values were fed continuously to the MCPs of the humanoid. Similarly, reconstructed hand gestures using the synthetic kinematic synergies were translated in a continuous manner to the MCPs. As the MCPs moved, they brought together the PIPs and DIPs to the target position from the reference posture. Each of the achieved targeted positions of the 28-hand gesture are shown in Figure 8.

One of the key limitations in this study was observed when using the MediaPipe hand landmark detection model. MediaPipe hand landmark detection model had difficulty to identify the different hand gestures when all the digits were close. Specifically, such difficulties were observed when identifying ‘M’, ‘N’, ‘O’ and ‘S’ hand gestures. It can be noted that in all these gestures, digits are extremely close to one another. This implies that the dots and lines of the hand landmark model were unable to clearly identify the overlaps especially with the usage of thumb flexion and extension. This might be because of the confidence parameter of the detection model kept to 0.5.

In Figure 6, it is evident that the reconstruction errors of gesture Seven and Eight are notably higher than the other hand gestures. This may potentially be attributed to the optimization algorithm attempting to accommodate all the different gestures using only six synthetic kinematic synergies. The complexity and variability of the gestures might pose challenges for the algorithm in finding an optimal fit within the limited set of synergies, thereby leading to higher reconstruction error.

Thus, the integration of Mitra with the synergy-based model not only enables the robot to learn the hand gestures from the test data but also facilitates the formation of a library of new synergies. This library is generated based on the new hand gestures demonstrated to Mitra apart from the selected hand gestures.

## 7 Conclusion

Improving the dexterity of humanoid robot hands enables robots excel in performing intricate tasks with precision, including surgical assistance and patient care, as well as aiding individuals with disabilities or elderly person in their daily activities. These humanoid robots, equipped with refined hand dexterity can play pivotal role across industries that involve the manipulation of objects and materials.

In this paper, we introduced a novel human-robot teaming approach for extracting synthetic kinematic synergies from end postures of hand gestures using a single RGB camera, MediaPipe framework, Gaussian functions and PCA. To the best of our knowledge, this study represents one of the first study in teaching a humanoid robot ASL hand gesture movements using kinematic synergies. Both offline and online models were developed in this study that incorporates 33 preconfigured ASL hand gestures covering a comprehensive range of finger joints. The current mapping of the synergies and reconstructed patterns to the robot may not be ideal, we aim to widen the scope by including more efficient mapping methods in the future. Enabling such synergy-based humanoid and robots have the potential to simplify the complexities associated with motion retargeting, offering promising applications in industrial robots and assistive robots.

## Data availability statement

The raw data supporting the conclusions of this article will be made available by the authors, without undue reservation.

## Ethics statement

The studies involving humans were approved by the University of Maryland Baltimore County 408-Selection of Optimal Control Signals for Human Machine Interfaces. The studies were conducted in accordance with the local legislation and institutional requirements. The participants provided their written informed consent to participate in this study.

## Author contributions

PO: Conceptualization, Data curation, Formal analysis, Investigation, Methodology, Project administration, Software, Validation, Visualization, Writing – original draft, Writing – review & editing. RV: Conceptualization, Data curation, Formal analysis, Funding acquisition, Investigation, Methodology, Project administration, Resources, Software, Supervision, Validation, Visualization, Writing – original draft, Writing – review & editing. DP: Formal analysis, Software, Visualization, Writing – review & editing. BK: Formal analysis, Software, Visualization, Writing – review & editing. AS: Writing – review & editing. NK: Writing – review & editing.
